# Immunoglobulin NGS enhance residual disease detection and prognosis in pediatric Ph+ acute lymphoblastic leukemia

**DOI:** 10.3389/fimmu.2025.1677013

**Published:** 2026-02-02

**Authors:** Lixian Chang, Jiao Chang, Beibei Zhao, Yun Gu, Yao Zou, Yumei Chen, Ye Guo, Xiaojuan Chen, Wenyu Yang, Yongjuan Duan, Tianyuan Hu, Xiaoming Liu, Min Ruan, Zefeng Lu, Shixin Lu, Xiaoxia Wang, Li Dong, Jinghua Wu, Yujiao Jia, Xiao Liu, Xiaofan Zhu, Li Zhang

**Affiliations:** 1 State Key Laboratory of Experimental Hematology, National Clinical Research Center for Blood Diseases, Haihe Laboratory of Cell Ecosystem, Institute of Hematology & Blood Diseases Hospital, Chinese Academy of Medical Sciences & Peking Union Medical College, Tianjin, China; 2 Tianjin Institutes of Health Science, Tianjin, China; 3 NeoImmune Co., Ltd., Shenzhen, China; 4 Shenzhen International Graduate School, Tsinghua University, Shenzhen, China

**Keywords:** clone evolution, Ig-NGS, immunoglobulin gene rearrangement, minimal residual disease, Ph+ B-ALL

## Abstract

In pediatric Philadelphia chromosome-positive acute lymphoblastic leukemia (Ph+ B-ALL), the clinical value of highly sensitive minimal residual disease (MRD) detection by immunoglobulin next-generation sequencing (Ig-NGS), and its role for tracking clonal evolution, remains inadequately characterized. In this study, we evaluated MRD in a cohort of pediatric Ph+ B-ALL patients using Ig-NGS in parallel with conventional methods, including flow cytometry (FCM) and BCR-ABL reverse transcription polymerase chain reaction (RT-PCR). Malignant clonal burden at diagnosis, MRD kinetics, and immunoglobulin heavy chain (IGH) clonal evolution were analyzed for their prognostic relevance. We observed that a lower percentage of malignant clonal cells detected by Ig-NGS at diagnosis was associated with improved relapse-free survival (RFS) (p < 0.01). Ig-NGS-derived pre-treatment malignant clone burden showed stronger association with relapse risk compared with FCM or RT-PCR. Furthermore, Ig-NGS MRD negativity at the end of induction (EOI) was associated with superior two-yeas RFS (p = 0.01), and Ig-NGS detected molecular relapse earlier than FCM or RT-PCR in some patients. Specific IGHV and IGHJ gene usage patterns and the extent of V-replacement clonal evolution at diagnosis were also correlated with prognosis. In summary, these findings suggested that Ig-NGS based MRD assessment may provide enhanced prognostic stratification and enable dynamic monitoring of clonal evolution in pediatric Ph+ B-ALL. Its integration into routine clinical practice may enhance early relapse prediction and support more precise risk-adapted therapeutic decisions.

## 1 Introduction

Philadelphia chromosome-positive B-cell acute lymphoblastic leukemia (Ph+ B-ALL), defined by the t(9;22) translocation resulting in constitutively activated BCR-ABL1 tyrosine kinase, accounts for approximately 3–4% of pediatric B-ALL cases (1, 2). Historically, pediatric Ph+ ALL was associated with a poor prognosis despite intensive chemotherapy and hematopoietic stem cell transplantation (HSCT). However, the introduction of tyrosine kinase inhibitors (TKI) has significantly improved treatment outcomes, including higher complete remission (CR) rates and prolonged survival. According to the national multicenter Chinese Children’s Cancer Group ALL-2015 (CCCG-ALL-2015) study, the five-year event-free survival (EFS) and overall survival (OS) rates of pediatric Ph+ ALL patients were 52.1% and 77.6%, respectively (3). More recently, the incorporation of immunotherapies, specifically blinatumomab-based treatment, has further improved prognosis; however, 20% of patients still experience recurrence (4). Therefore, further research is required to develop approaches for the early identification of relapse-related factors and genetic features.

Minimal residual disease (MRD) monitoring plays a critical role in predicting relapse and guiding risk-adapted treatment strategy. Current MRD detection methods include multi-parametric flow cytometry (FCM), reverse transcription polymerase chain reaction (RT-PCR), and immunoglobulin gene rearrangement next-generation sequencing (Ig-NGS), each of which has distinct advantage and limitation, as summarized in prior reviews (5–7). FCM, while widely used, has a relatively limited sensitivity (10^-4^) compared with the other two molecular methods. RT-PCR offers higher sensitivity (10^-5^) but is restricted to quantify BCR::ABL transcripts and is therefore applicable only in Ph+ ALL. Ig-NGS enables tracking of leukemia-specific immunoglobulin gene rearrangements with a sensitivity of up to 10^-6^, which is 1–2 log improvement over conventional FCM and RT-PCR. Despite its potential, comprehensive studies evaluating the prognosis significance of Ig-NGS and its concordance with FCM and RT-PCR in pediatric Ph+ ALL remain limited. The aim of this study is to establish an improved MRD monitoring framework by evaluating the clinical utility of Ig-NGS and to define MRD-driven risk stratification strategies in pediatric Ph+ B-ALL.

B-cell clonal evolution is a key mechanism in the pathogenesis and relapse of B-ALL. Relapse may originated from subclones present at diagnosis or from newly emerged subclones under treatment pressure (8). Ig-NGS offers an effective approach to trace clonal dynamics, enabling the detection of clonal evolution events, such as V-replacement or subclonal emergence, which may inform therapeutic response and long term prognosis (9, 10). Therefore, another aim of this study is to investigate immunoglobulin heavy chain (IGH) rearrangement characteristic and the clonal evolution patterns in Ph+ B-ALL patients at diagnosis, during treatment, and at relapse using Ig-NGS. Our findings will provide a perspective for understanding the biological basis of relapse in pediatric Ph+ B-ALL.

## 2 Materials and methods

### 2.1 Patients and samples

Pediatric patients with Ph+ B-ALL were enrolled between May 2015 and April 2020 at the Hematology Hospital of the Chinese Academy of Medical Sciences. The diagnosis of Ph+ B-ALL was based on bone marrow cell morphology and the detection of the BCR::ABL fusion gene. Eligible patients received frontline therapy according to the Chinese Children’s Cancer Group ALL-2015 (CCCG-ALL-2015) protocol, which includes chemotherapy combined with a BCR-ABL1 TKI regimen (ChiCTR-IPR-14005706) (4). Informed consent was obtained from the parents or guardians of all participants.

Relapse-free survival (RFS) was defined for patients who achieved complete remission (CR) and was measured from the date of CR to the date of relapse or the last available follow-up date.

### 2.2 FCM-based and BCR-ABL RT-PCR-based MRD assessment

MRD was assessed using bone marrow (BM) samples via FCM and BCR-ABL RT-PCR at the Hematology Hospital of the Chinese Academy of Medical Sciences.

### 2.3 Ig-NGS based MRD assessment

High-quality genomic (gDNA) was extracted from frozen BM or peripheral blood (PB) samples using the HiPure Blood DNA Mini Kit (MAGEN; Cat. no. D3111-03) and analyzed with the NEOMRD^®^ assay (Neoimmune, Shenzhen, China). Briefly, two rounds of PCR were performed to enrich the V(D) J-rearranged sequences from the IGH, IGK, and IGL loci. For diagnostic samples, 500 ng of gDNA was used, and for post-treatment samples, 500–20,000 ng of gDNA was used.

The first round PCR was a multiplex PCR comprising 28 cycles, using forward primers targeting the variable (V) gene segments and reverse primers targeting joining (J) gene segments of the IGH, IGK, and IGL loci. The second step was universal PCR using a universal primer and 12 cycles, which added the whole adaptor sequence to generate libraries for sequencing on the DNBSEQ-T7 (paired-end 150 bp reads).

The raw sequencing reads were processed using an in-house-developed bioinformatics pipeline (NEOIMONITOR); To eliminate artifacts, the rearranged clone sequences in a sample with a frequencies below 3 per million were excluded. Dominant clone sequences were identified in the diagnostic sample and tracked in the follow-up samples if they met the following criteria: 1) the clone proportion of IGH, IGK or IGL was > 3%, 2) the percentage of nucleated cells was > 0.2%, and 3) the clone sequences exhibited a discontinuous distribution.

Once suitable dominant clone sequences were identified, they were tracked in the post-treatment samples. For the IGH clones, the perfected matched sequences and sequences with up to two nucleotide mismatches to the dominant sequences were used to calculate the MRD; for the IGK and IGL clones, only exact matches were used. The final MRD level for each sample was determined based on the chain hierarchy of IGH, IGL, and then IGK, which was calculated by summing the nucleated cells percentages in the selected chain. The analytical sensitivity of the assay can reached 10^-6^, depending on the total DNA input.

### 2.4 V-replacement analysis

V-replacement events were defined as clones sharing the same D-J rearrangements with the dominant IGH clones and exhibiting at least 50% sequence identity at the 3′ end of the CDR3, but utilizing a different upstream V gene segment as annotated by the IMGT database (**Supplementary Figure S1**).

For normalization, 50000 sequencing reads were randomly subsampled from each sample. The total number of evolved clones per patient was determined by summing all V-replacement clones derived from all the dominant IGH clones identified at baseline.

### 2.5 Statistical analysis

Fisher’s exact test was used for categorical variables, while Wilcoxon rank-sum test for continuous variables. RFS curves were estimated using the Kaplan–Meier method and compared using the log-rank test. All statistical analyses and data visualization were performed using R software (version 4.1.2), with the following packages: ggplot2, survival, survminer, pROC and dplyr. A two-tailed p-value < 0.05 was considered statistically significant.

## 3 Results

### 3.1 Patient clinical characteristics and distribution patterns of Ig clonal rearrangements

A total of 55 pediatric Ph+ B-ALL patients were enrolled in this study. Among them, two patients discontinued treatment due to financial constraints, one patient died during induction therapy, and three patients had insufficient BM samples for analysis post-induction. The remaining 49 patients were included in the final evaluation, among whom 48 (98%) exhibited at least one trackable Ig clonal rearrangement and were included in subsequent analyses.

Baseline characteristics of these patients are summarized in **Table 1**. Briefly, this cohort comprised 28 male and 20 female patients, with a median age of 8.4 years (range, 2.6–15.8 years). The median follow-up duration was 50 months (range, 6–98 months). By the last follow-up, 35% of the patients had experienced relapse, and 17% had died.

**Table 1:** Baseline characteristics of patients (n = 48).

Characteristics	Number	*%*
Sex		
Male	28	58.3
Female	20	41.7
Age (years)		
1 to 5	6	13.5
5 to 10	22	45.8
≥ 10	20	41.7
Hepatomegaly		
No	31	64.6
Yes	17	35.4
Splenomegaly		
No	19	39.6
Yes	29	60.4
CNSL		
CNSL1	44	91.7
CNSL2	4	8.3
Clinical outcome		
Relapse	17	35.4
Non-relapse	31	64.6

IGH clonal rearrangements were detected in 95.8% of the patients, with most (75%) having one (31.2%) or two (43.8%) IGH clones. IGK clonal rearrangements were present in 43.8% of patients with 85.7% of those exhibiting a single IGK clones. IGL clonal rearrangements were less frequent, observed only in 25% of patients, with 83.3% exhibiting a single IGL clone. In total, 89 IGH, 25 IGK, and 14 IGL clonal rearrangements were identified. The distribution of Ig clones across patients is detailed in **Supplementary Figure S2** and **Supplementary Table S1**.

### 3.2 Proportion of malignant clonal cells detected by Ig-NGS before treatment as a predictor of relapse risk

In this cohort, the median proportion of malignant clonal cells prior to treatment was 35.87% (range: 0.23–99.22%) as assessed by Ig-NGS. In comparison, the median value were 91.74% (range: 0.16–153.12%) by RT-PCR, and 81.90% (range: 36.40–94.60%) by FCM.

**Figure 1:**
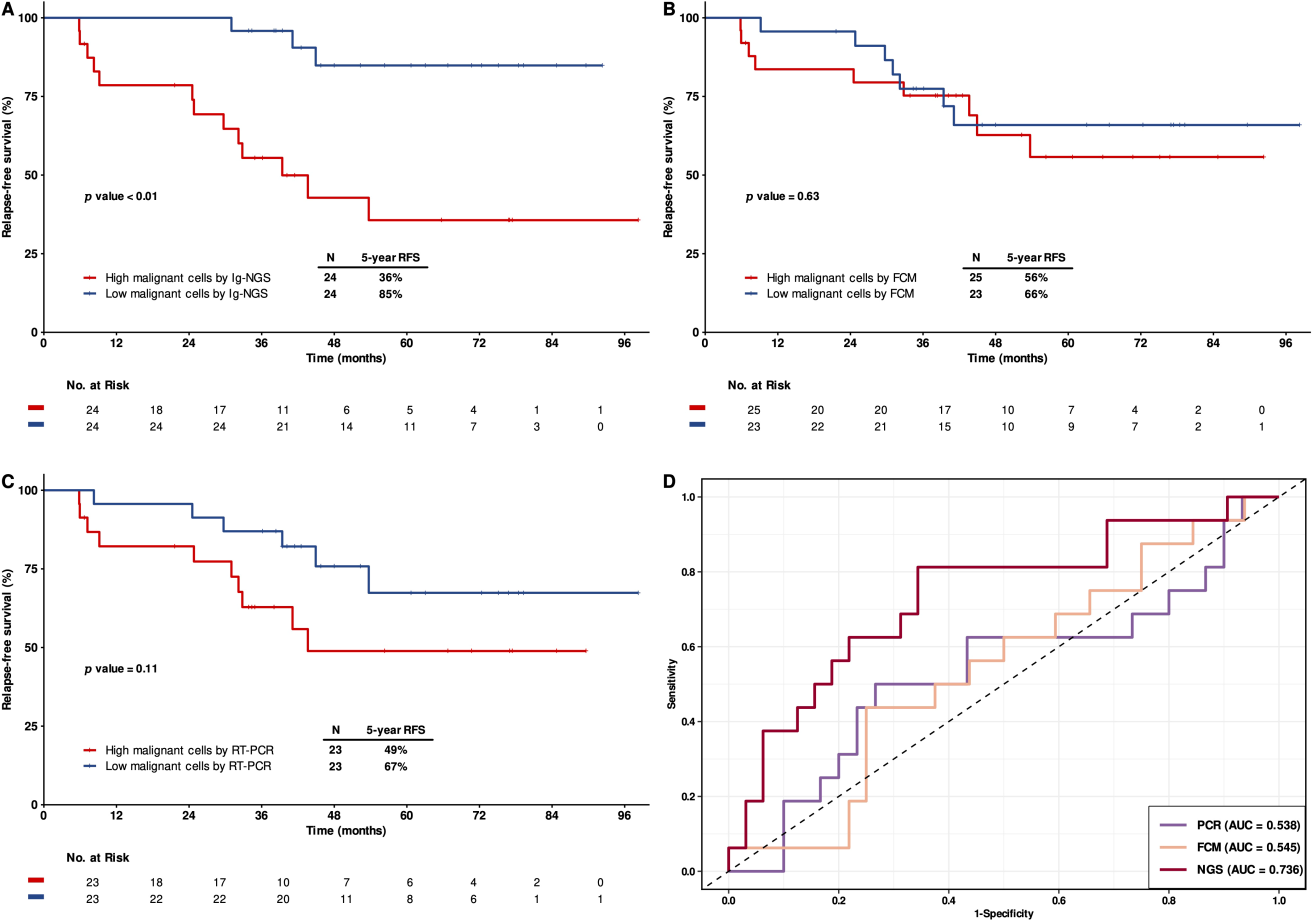
Prognostic relevance of malignant clonal cells burden at diagnosis. **(A-C)** Kaplan-Meier estimates of relapse-free survival (RFS) stratified by baseline malignant clonal cell proportion as assessed by Ig-NGS **(A)**, FCM **(B)** and RT-PCR **(C)**. **(D)** Receiver operating characteristic (ROC) curves to evaluate the predictive performance of the three methods in assessing relapse risk based on initial malignant cell burden.

The relapse-free survival (RFS) of patients with a lower proportion of malignant clonal cells by Ig-NGS (below the median) was significantly superior compared to those with higher level (5-year RFS: 85% vs. 36%, p < 0.01; **Figure 1A**). In contrast, no significant difference in relapse risk was observed when stratifying patients based on malignant cells burden assessed by FCM (5-year RFS: 66% vs. 56%, p = 0.63; **Figure 1B**) or RT-PCR (5-year RFS: 67% vs. 49%, p = 0.11; **Figure 1C**).

Receiver operating characteristic (ROC) curves analysis was conducted to evaluate the predictive value of baseline malignant cell burden determined by each method. Ig-NGS demonstrated the highest area under the curve (AUC), indicating superior diagnostic performance for relapse prediction (**Figure 1D**).

### 3.3 IGHV and IGHJ gene usage of malignant Ig clones and their association with prognosis

**Figure 2:**
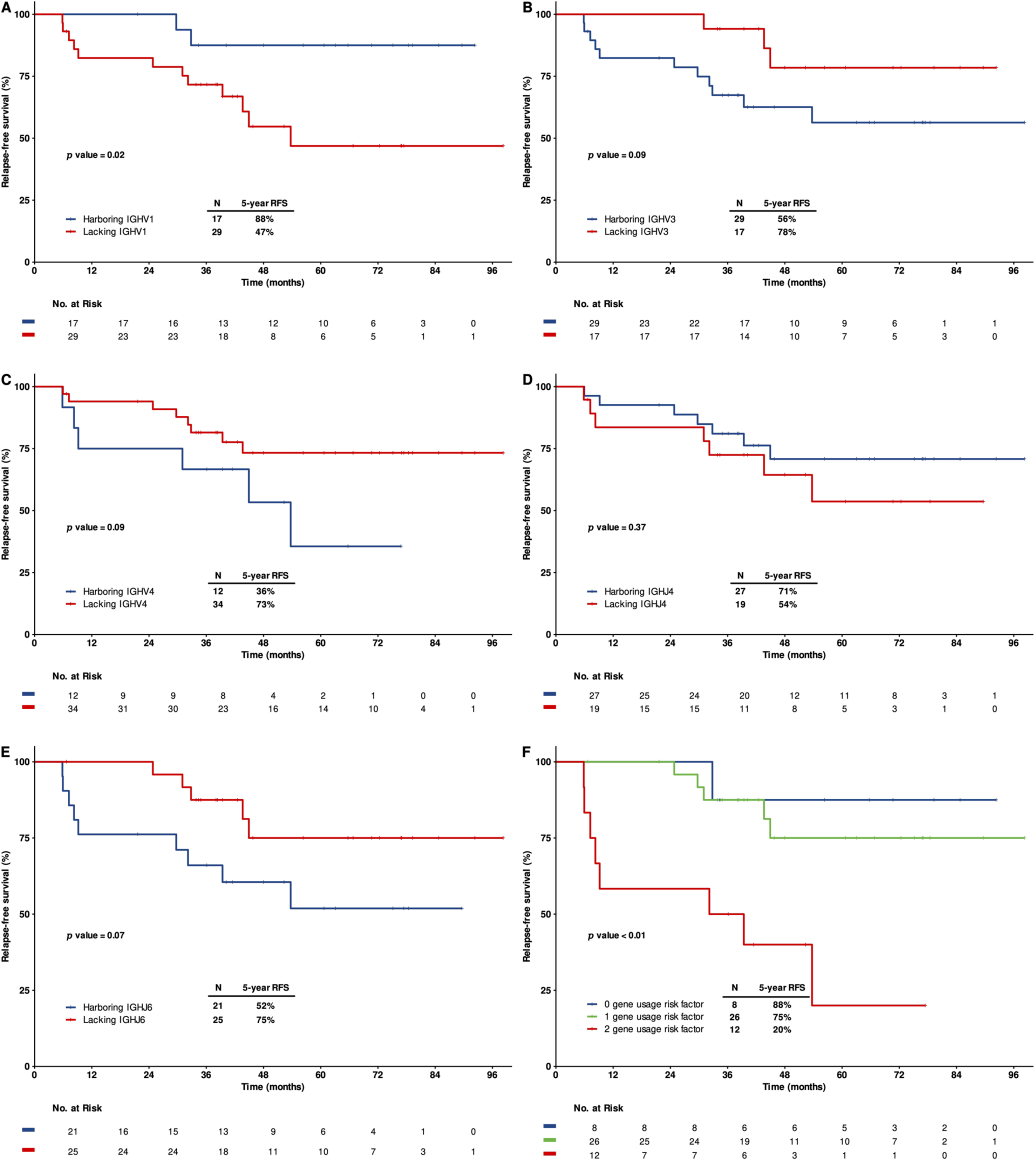
Prognostic significance of IGHV and IGHJ gene usage of malignant IGH clones. Kaplan-Meier curves showing 5-year relapse-free survival (RFS) based on the specific immunoglobulin gene segments in dominant IGH clone: **(A)** IGHV1, **(B)** IGHV3, **(C)** IGHV4, **(D)** IGHJ4, **(E)** IGHJ6 and **(F)** different combined usage of IGHV1gene and IGHJ6 gene.

To investigate the prognosis relevance of malignant Ig clones, we analyzed the IGHV and IGHJ gene segment usage of malignant Ig clones and their association with RFS. Among patients with detectable IGH rearrangements, the most frequently used IGHV gene families in clonal Ig rearrangements were IGHV3 (63%), IGHV1 (37%), and IGHV4 (26%) (**Supplementary Figure S3A**). Notably, patients harboring IGHV1 gene demonstrated significantly improved five-year RFS compared with those lacking IGHV1 gene (88% vs. 47%, p = 0.02; **Figure 2A**). In contrast, the presence of IGHV3 or IGHV4 genes was not associated with a significant difference in five-year RFS (**Figures 2B, C**).

The most commonly used IGHJ genes were IGHJ4 (59%) and IGHJ6 (46%) (**Supplementary Figure S3B**). While IGHJ4 gene usage was not associated with significant differences in prognosis (p = 0.37; **Figure 2D**), patients harboring IGHJ6 gene showed a trend towards inferior five-years RFS (p = 0.07; **Figure 2E**). The combination of lacking IGHV1 gene and possessing IGHJ6 gene was associated with the poorest prognosis, with a five-year RFS of only 20% (**Figure 2F**). Analysis of the IGHV gene usage patterns between relapsed and non-relapsed patient did not reveal distinct locus-specific biases (**Supplementary Figure S4**).

### 3.4 Concordance of MRD detection methods and comparative performance in risk stratification

**Figure 3:**
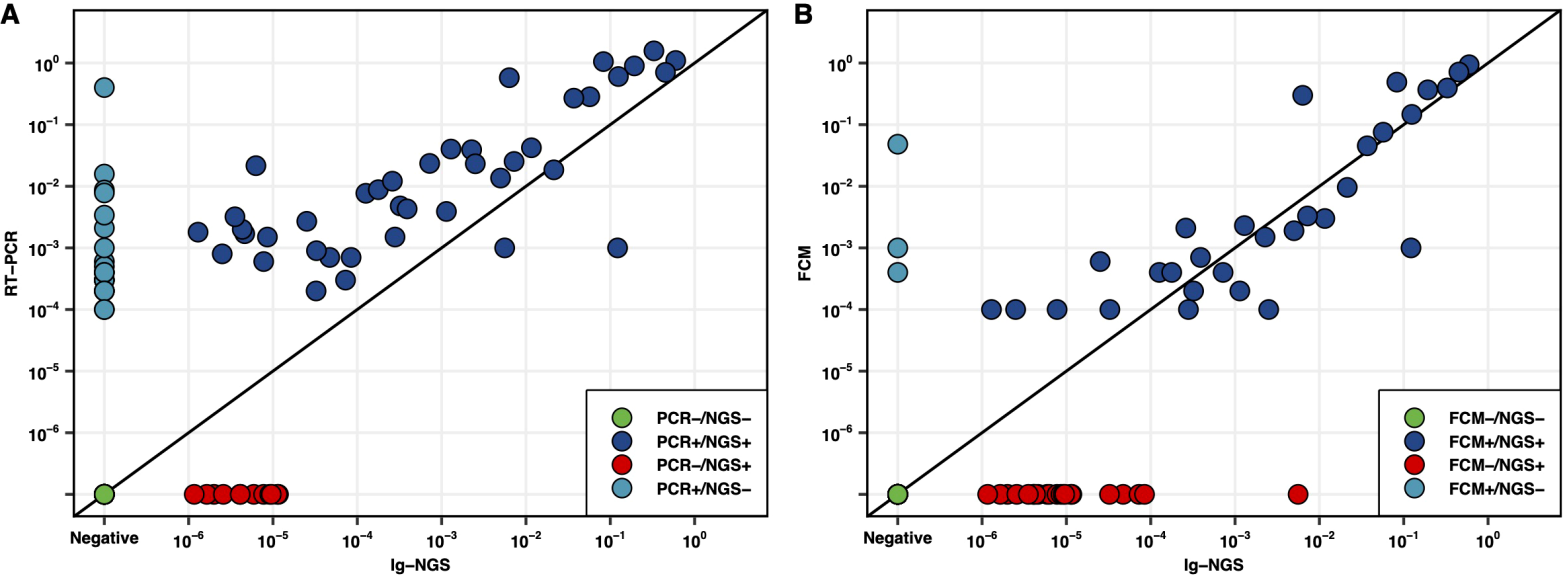
Comparison of the MRD detection methods. **(A)** Scatterplot comparing MRD levels as measured by RT-PCR and Ig-NGS. **(B)** Scatterplot comparing MRD levels as measured by FCM and Ig-NGS.

MRD was concurrently assessed by Ig-NGS, FCM and RT-PCR in 100 post-treatment samples. Among those sample, 32% showed discordant results between RT-PCR and Ig-NGS (20 samples were RT-PCR+/NGS- and 12 were RT-PCR-/NGS+; **Figure 3A**). Similarity, 25% of the samples showed discrepancies between FCM and Ig-NGS with 3 FCM+/NGS- and 22 were FCM-/NGS+ (**Figure 3B**). Overall, Ig-NGS demonstrated greater concordance with FCM than with RT-PCR.

**Figure 4:**
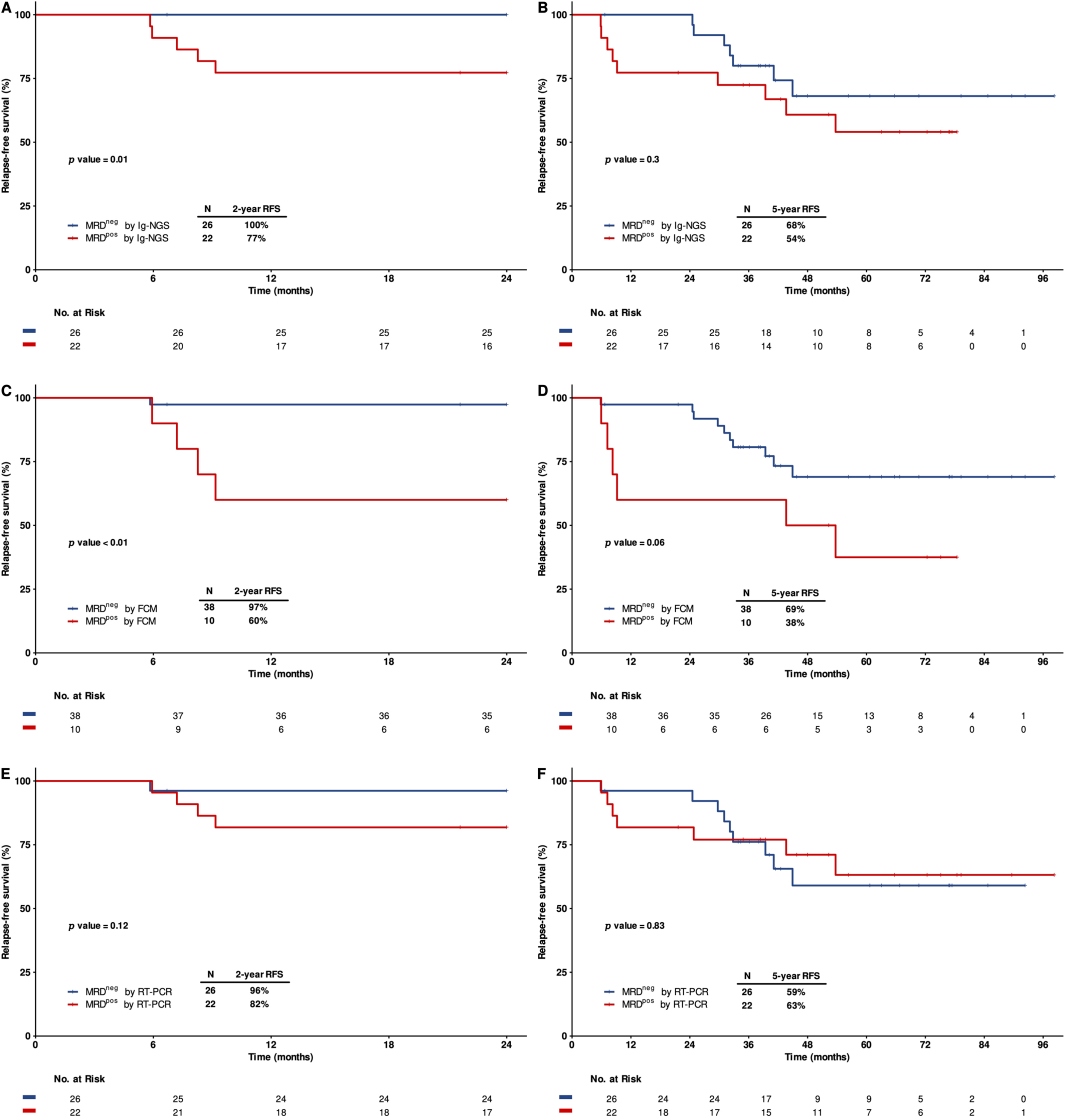
Prognostic value of MRD status at the end of induction therapy (EOI). Kaplan-Meier estimates relapse-free survival (RFS) stratified by MRD status at EOI: **(A, B)** Two-years and five- years RFS, respectively, based on Ig-NGS. **(C, D)** Two-year and five-years RFS, respectively, based on FCM. **(E, F)** Two-years and five-years RFS, respectively, based on RT-PCR.

At the end of induction therapy (EOI), 54% of patients achieved Ig-NGS MRD negativity, and of these patients, none relapsed within two years. In contrast, 23% of the patients who remained Ig-NGS MRD positive at EOI relapsed within two years. Two-year RFS was significantly higher among Ig-NGS MRD negative patients compared to those who were MRD positive (100% vs. 77%, p = 0.01; **Figure 4A**). However, by five years, the difference in RFS between the two groups was no longer statistically significant (68% vs. 54%, p = 0.3; **Figure 4B**).

When assessed by FCM, 79% of patients achieved MRD negativity at EOI, and these patients had improved two-years RFS compared to those who were MRD positive (97% vs. 60%, p < 0.01; **Figure 4C**). However, one patient who was FCM MRD negative at EOI relapsed within six months. Five-years RFS also trended higher among FCM MRD negative patients but did not reach statistical significance (69% vs. 38%, p = 0.06; **Figure 4D**). MRD assessment by RT-PCR are not effective in predicting relapse at EOI, with two-year RFS of 96% and 82% (p = 0.12), five-year RFS of 59% and 63% (p = 0.83) for RT-PCR MRD negative and positive patients (**Figures 4E, F**).

When combining MRD results from multiple methods at EOI, patients who were dual-negative by FCM and Ig-NGS had the best two-years RFS, whereas patients who were dual-positive had the worst outcomes (**Supplementary Figure S5A**). Patients with FCM-/NGS+ status had an increased relapse risk compared to dual-negative patients (**Supplementary Figure S5A**). Notably, among patients with PCR+/NGS-, no relapses were observed within two years, suggesting higher false positive of RT-PCR in MRD detection (**Supplementary Figure S5C**). However, the predictive accuracy of RT-PCR or FCM combined with Ig-NGS remained limited at the five-year (**Supplementary Figures S5B, D**).

Interestingly, patients who were MRD double-negative by RT-PCR and FCM paradoxically exhibited worse long-term outcomes than those who were RT-PCR-positive but FCM-negative (two-year RFS, 96% vs. 100%; five-year RFS, 59% vs. 91%; **Supplementary Figures S5E, F**), which also suggesting higher false positive of RT-PCR in MRD detection.

### 3.5 Ig-NGS MRD serves as an early predictor of relapse

Of the 17 patients who relapsed, 15 (88.2%) exhibited re-emergence of the dominant Ig clones identified at diagnosis in their relapsed samples. In one patient, although the original dominant clones were not detected, five new dominant clones were identified at relapse. In another case, neither the original nor any new dominant clones were detected in the relapsed sample.

**Figure 5:**
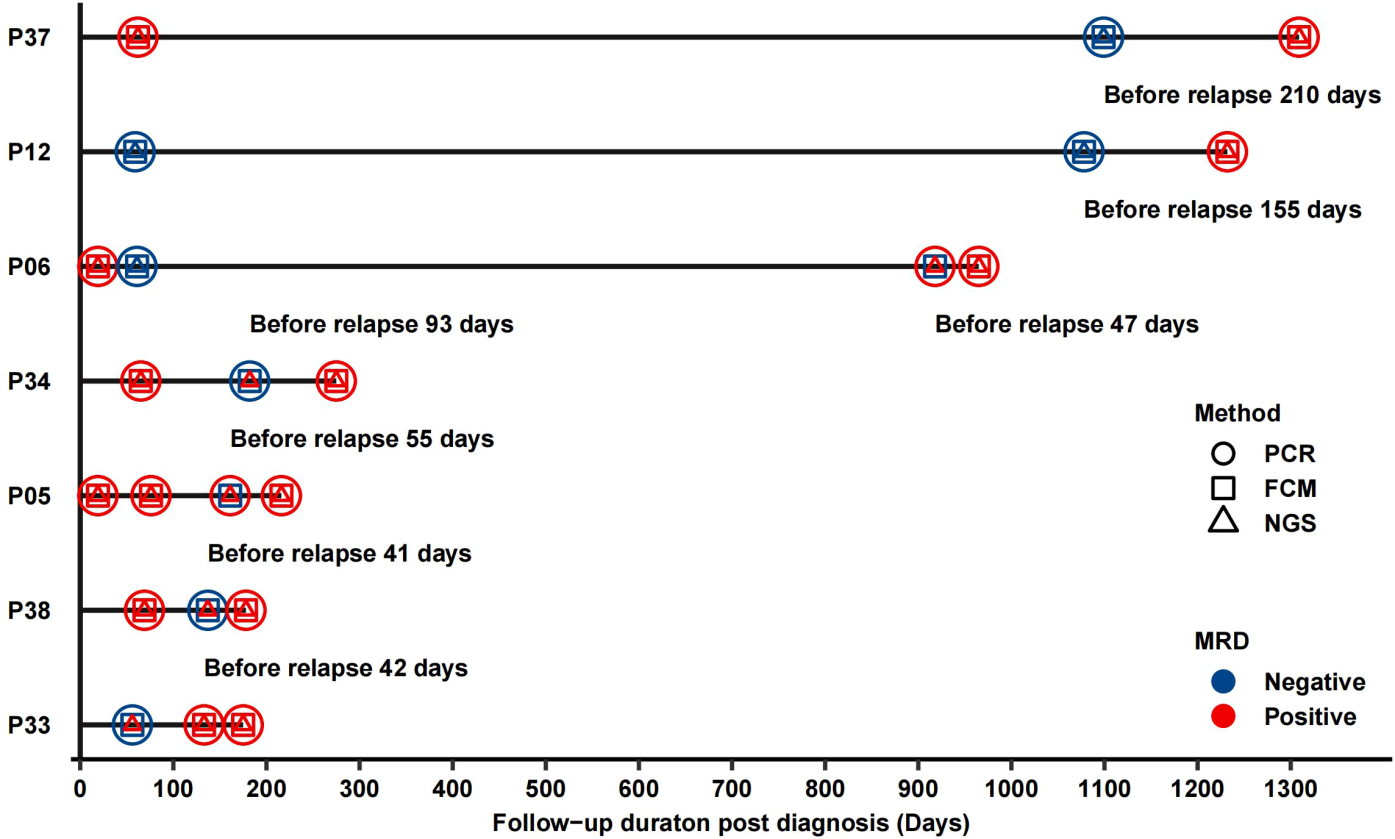
MRD dynamics preceding clinical relapse. The MRD status detected by Ig-NGS, FCM, and RT-PCR in seven patients who underwent MRD monitoring within one year prior to relapse.

To assess the ability of MRD methods in predicting relapse in advance, we analyzed the longitudinal MRD results for seven patients who had MRD testing performed within one year prior to clinical relapse (other than at EOI). In five of the seven patients, the last MRD test was conducted within 150 days before relapse. Ig-NGS detected positive MRD in all five samples, demonstrated its early predictive capability. In contrast, only three of the five patients showed positive RT-PCR results, and just one showed positive FCM results (**Figure 5**). For the remaining two patients, the last MRD test was performed more than 150 days before relapse, and all three methods yielded negative MRD results at that time point. These observations underscore the superior sensitivity of Ig-NGS for detecting molecular relapse ahead of clinical progression and suggest its potential utility for early therapeutic intervention.

### 3.6 Clonal evolution predicts patient prognosis

Clonal evolution of malignant B cells is considered a pivotal process in ALL. To evaluate the clinical relevance of clonal evolution, we analyzed the emergence of V-replacement clones in patients with detectable IGH rearrangements at diagnosis (D0) and at EOI. At D0, 70% of the patients had V-replacement evolutionary clones (median: 14; range: 1–1728), while 30% of patients had such evolutionary clones detectable at EOI (median: 27; range: 1–2129) (**Supplementary Table S2**).

**Figure 6:**
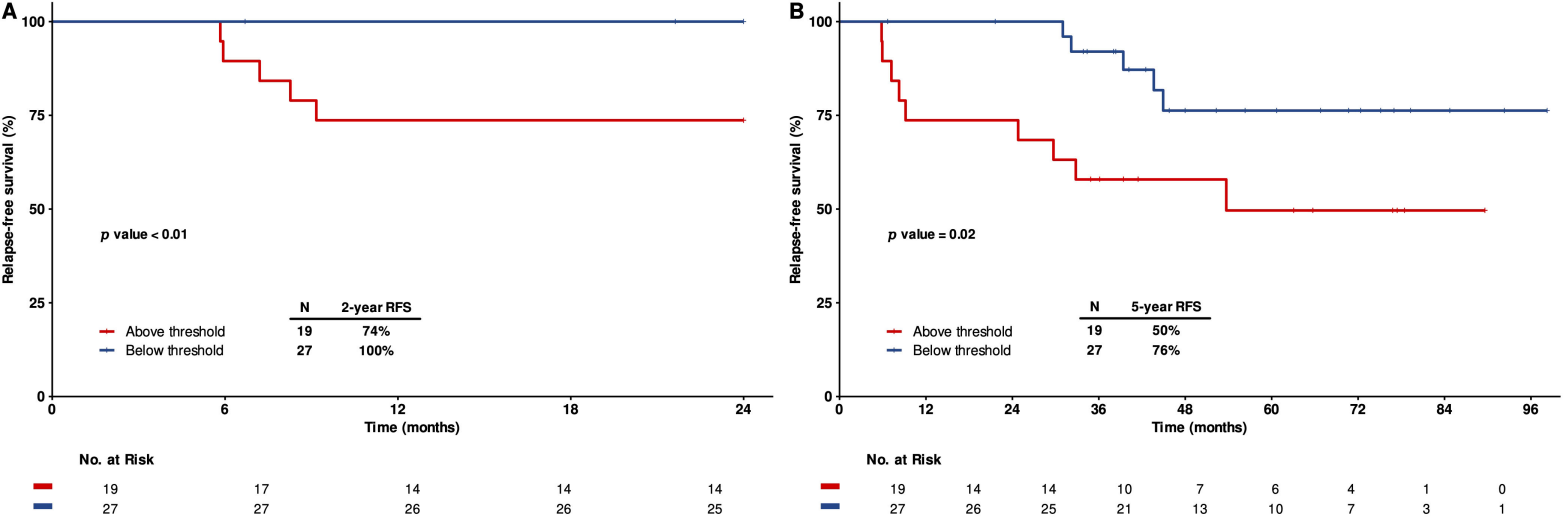
Prognostic value of clonal evolution assessed by Ig-NGS at diagnosis. Kaplan-Meier estimates of relapse-free survival (RFS) stratified by the number of V-replacement clones at diagnosis (D0). **(A)** two-years RFS, **(B)** five-years RFS.

To assess the prognostic value of clonal evolution, we applied ROC curves analysis to determine the optimal cutoff values for stratifying patients outcomes. At baseline (D0), a threshold of 5.5 evolved clones yielded the highest area under the curve (AUC = 0.648), with a sensitivity of 64% and a specificity of 69%. Patients with fewer than 5.5 V-replacement clones at D0 demonstrated significantly better survival outcomes comparing to those with higher clone counts. Specifically, two-year RFS was 100% for the low-evolution group compared to 74% for the high-evolution group (p < 0.01, **Figure 6A**), and five-year RFS was 76% versus 50%, respectively (p = 0.03, **Figure 6B**).

At EOI, a cutoff of 32 evolved clones yielded the highest AUC (0.602), although the association between high clonal evolution and relapse risk at this time point was less pronounced (**Supplementary Figure S6**).

These results suggested that the extent of clonal evolution, particularly at diagnosis, is associated with long-term prognosis in pediatric Ph+ B-ALL, and may provide a useful biomarker for risk stratification.

## 4 Discussion

Risk stratification at diagnosis and MRD guided post-treatment management are pivotal to optimize outcomes in patients with acute lymphoblastic leukemia. Contemporary treatment protocols emphasize integrating clinical, genetic, and molecular features at diagnosis to classify patients into risk groups, while post-induction MRD assessment evaluates treatment response and refines treatment intensity to balance efficacy and toxicity.

The discordance of MRD results detected by different methods was reported by a review article (11). Short et al. reported that the MRD results obtained using RT-PCR and NGS were discordant for 32% of Ph+ ALL patients (12). Similarity, in our study, 33% of patients showed discordance between RT-PCR and Ig-NGS, and Ig-NGS MRD at EOI demonstrated superior ability to predict two-years relapse compared to RT-PCR. The poor performance of RT-PCR MRD in predicting relapse may be due to the presence of BCR::ABL transcripts in non-leukemia cells, such as CML-like hematopoietic stem cell or myeloid cells, which are not relevant to active ALL (13). However, at the five years timepoint, the predictive value of EOI MRD (regardless of detection method) declined substantially in this Ph+ B-ALL patients group. Notably, although none of the Ig-NGS MRD negative patients relapsed within two years, 26.9% did relapse during extended follow-up, especially after the end of chemotherapy. These finding suggest that prolonging tyrosinase inhibitors maintenance time beyond chemotherapy cessation may be necessary to prevent late relapse. Importantly, Ig-NGS proved capable of detecting molecular relapse earlier than either FCM or RT-PCR. Among five patients who had MRD testing performed 150 days before relapse, all were Ig-NGS MRD positive, while only three were RT-PCR positive, and one FCM positive. Prior studies have shown that Ig-NGS also outperforms FCM in predicting relapse in patients who experienced CAR-T therapy (14). This early detection window could provide a critical opportunity for preemptive intervention. Together, those finding support routine longitudinal MRD monitoring using Ig-NGS for up to five years following treatment, particularly in high risk subgroups. If MRD is positive, immunotherapy, such as Blinatumomab (a CD19 directed bispecific T-cell-engager), should be considered to eliminate residual tumor cells and reduce relapse risk (15–17).

In addition to bone marrow (BM), peripheral blood (PB) has shown strong correlation with BM MRD levels in adult ALL patients, with reported correlation coefficients as r = 0.87 (p < 0.001) (18). In Pulsipher’s study, they discovered that although PB MRD is typically lower than BM MRD detected by the same method, PB based Ig-NGS may detect disease more sensitive than BM based FCM (14). There, PB sample via Ig-NGS may serve a minimally invasive alternative for long term surveillance, especially after achievement of BM MRD negativity (19, 20).

Previous research had showed that the predominant IGH sequences are associated with good five-year RFS rates among pediatric patients with high-risk B-ALL who lacked favorable cytogenetics (21). Interestingly, our study found that a lower baseline malignant clone proportions detected by Ig-NGS was associated with superior relapse-free survival. To our knowledge, this is the first report demonstrating such an association in ALL. Given that all patients exhibited similarly high leukemia burden by FCM at diagnosis, this finding is unlikely to reflect differences in bulk tumor load. One possible biological explanation is that a lower dominant clone proportion reflects greater intratumoral clonal heterogeneity, in which single clone did not possess a strong proliferation or survival advantage. In addition, although the Ig-NGS assay used in this study is analytically validated for absolute quantification, unrecognized technical or cohort-specific factors cannot be completely excluded given the relatively limited sample size.

The use of specific IGHV and IGHJ genes segments in tumor IGH rearrangements has been demonstrated (22–24) and associated with prognosis in other B-lineage malignancies (25–28), but has not been well-characterized in pediatric Ph+ ALL. For example, IGHV3–21 usage has been associated with shorter overall survival in chronic lymphocytic leukemia (25–27), while IGHV4–34 and IGHV5 have been identified as a predictive marker of histological transformation into aggressive lymphoma in follicular lymphomas (22, 28). In our study, we observed that patients with IGHV1 had better outcomes, while IGHJ6 usage was associated with inferior RFS. The worse outcomes were observed in patients lacking IGHV1 but with IGHJ6 genes. These findings suggest that specific IGHV and IGHJ gene segment usage may provide additional prognostic information in pediatric Ph+ B-ALL.

The mechanisms of clonal evolution in IGH mainly include VH replacement and the ongoing recombination of D-JH rearrangement with multiple VH fragments (29, 30). Some studies have raised the possible clinical relevance of IGH clonal evolution in B-ALL patients (21, 30, 31). In our study, we focused on the V-replacement evolution, and investigated the clinical relevance of V-replacement clones at diagnosis and EOI. Our data showed that, at diagnosis, 70% (32/46) of the patients harboring V-replacement clones, and a lower number of such clones was associated with improved two- and five-year survival. This contrasts with the finding by Fries et al., who discovered that while V-DJ subclonal evolution is prevalent in B-ALL, it was not independently associated with prognosis (21). Nevertheless, our data suggest that the degree of IGH clonal evolution at baseline reflects underlying genomic instability and may serve as an independent prognostic indicator.

This study has several limitations that should been acknowledged. First, its retrospective, single-center design may introduce some bias. Second, the sample size was relatively modest, which restricts the statistical power, particularly for multiple subgroup analyses such as those based on IGHV and IGHJ gene usage. As a result, some associations should be interpreted with caution. Third, the finding may be influenced by the distinct biological characteristic of Ph+ ALL, which may limit generalizability to other ALL subtypes. Therefore, multicenter studies with larger sample sizes and more comprehensive clinical features are needed to validate these discoveries. In addition, although Ig-NGS demonstrates higher sensitivity and analytical robustness, its broader clinical implementation may be constrained by technical complexity, cost considerations and the need of standardized bioinformatic pipeline and quality control procedures.

## 5 Conclusion

This study demonstrates that Ig-NGS provides added sensitivity and prognostic value for MRD monitoring and clonal evolution analysis in Ph+ B-ALL. Compared with conventional methods such as flow cytometry and RT-PCR, Ig-NGS enables earlier molecular detection of relapse in a subset of patients, particularly within the first two years after induction therapy. Importantly, the percentage of malignant clonal cells at diagnosis, as well as the degree of V-replacement clonal evolution, were associated with long-term relapse risk, indicating their potential as biomarkers for risk stratification. Additionally, specific IGHV and IGHJ gene usage patterns were correlated with clinical outcomes, offering further insight into disease biology.

Overall, these findings indicate that Ig-NGS may serve as a comprehensive tool for MRD surveillance and for characterizing B-cell clonal dynamics in Ph+ B-ALL. Integrating Ig-NGS into routine clinical practice may facilitate more precise, risk-adapted treatment strategies and ultimately improve outcomes for children with Ph+ B-ALL.

## Data Availability

The raw sequencing data generated in this study are available under restricted access in the Genome Sequence Archive (GSA) for Human under bioProject PRJCA055449 (https://ngdc.cncb.ac.cn/gsa-human/browse/HRA016085). Access to the data is restricted to protect individual genetic information; however, the data are available from the corresponding author upon reasonable request.
