# Supplementary material for: Immunoglobulin NGS enhance residual disease detection and prognosis in pediatric Ph+ acute lymphoblastic leukemia
Source: Front Immunol. 2026 Feb 2;16:1677013. doi: 10.3389/fimmu.2025.1677013 (PMC12907345; doi:10.3389/fimmu.2025.1677013)
Supplement: Supplementary file 1 [file DataSheet1.docx]

**Supplementary Figures**


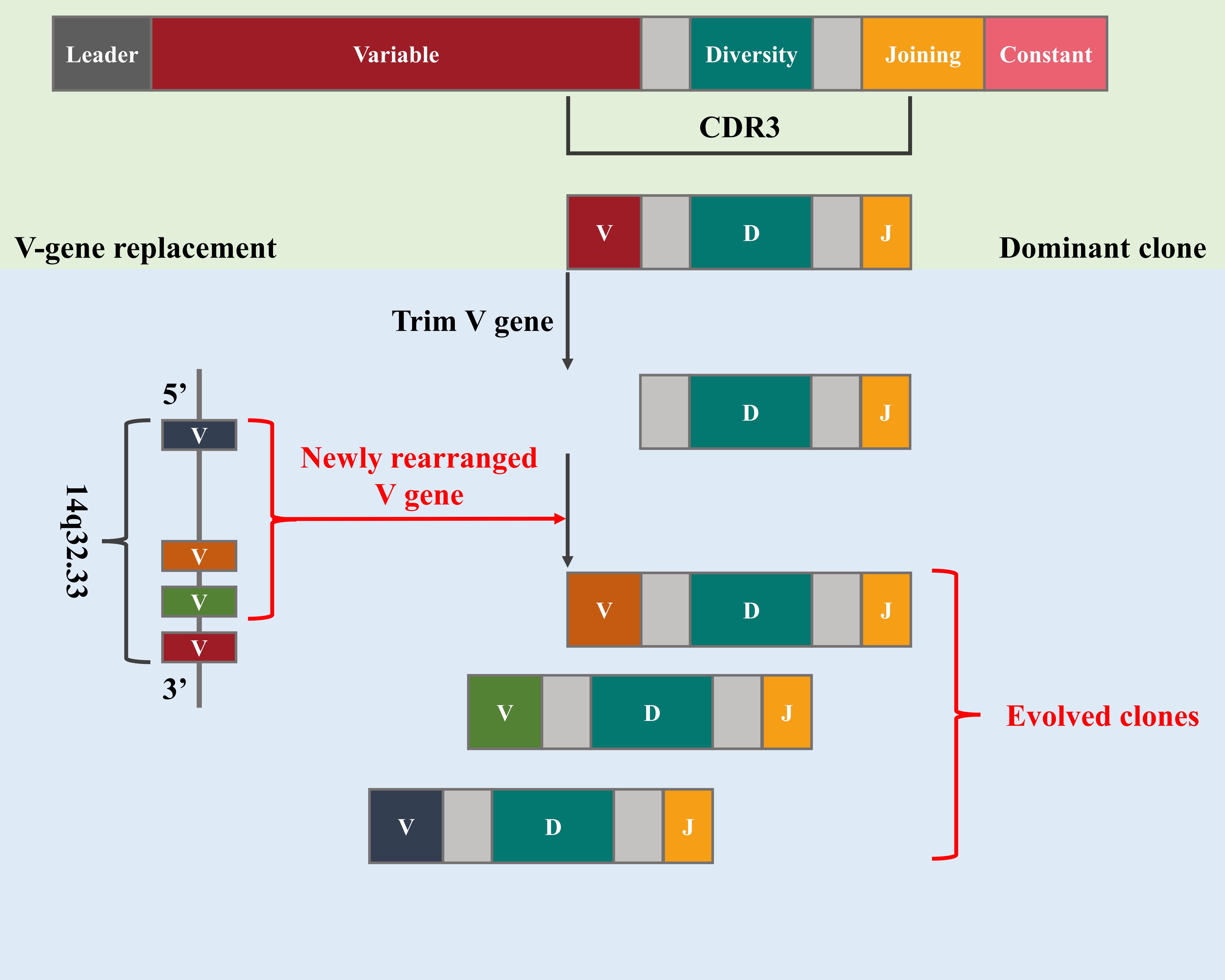


**Figure S1.** V-replacement clonal evolution.


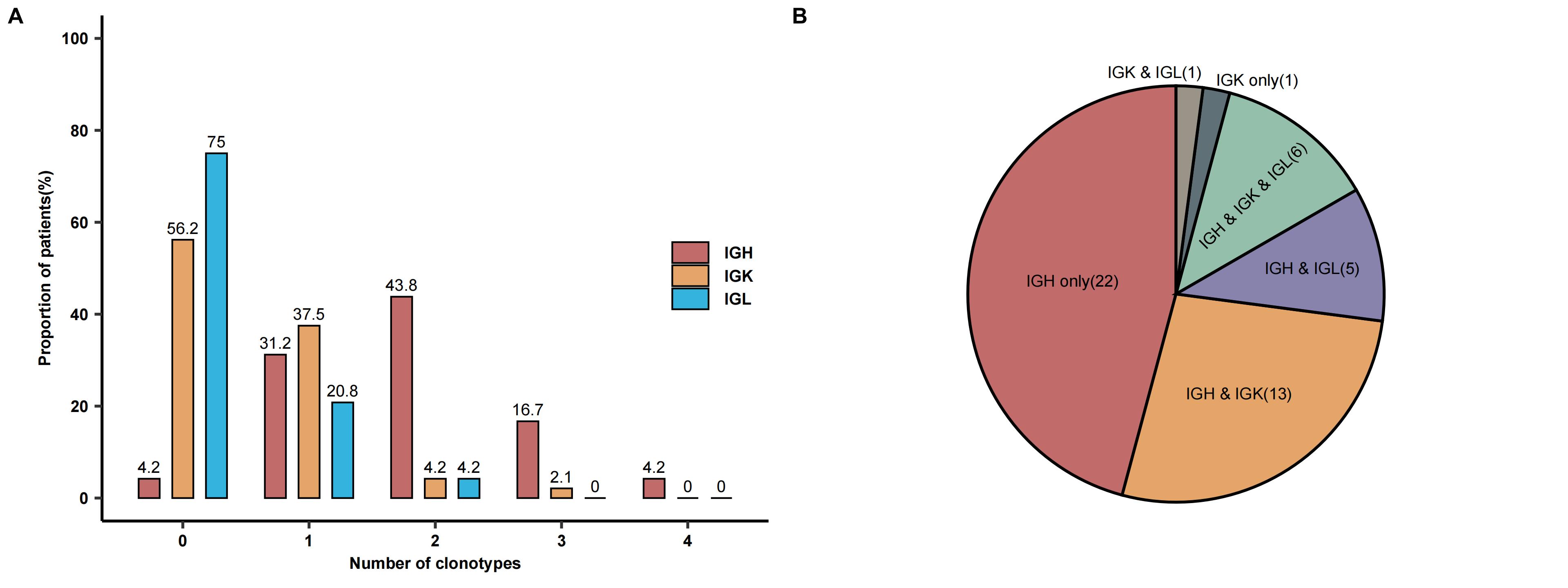


**Figure S2.** **Distribution of identified Ig clone**. (A) Distribution of clone counts for IGH, IGK and IGL loci among cohort. (B) Number of patients with different combinations of Ig clone types.


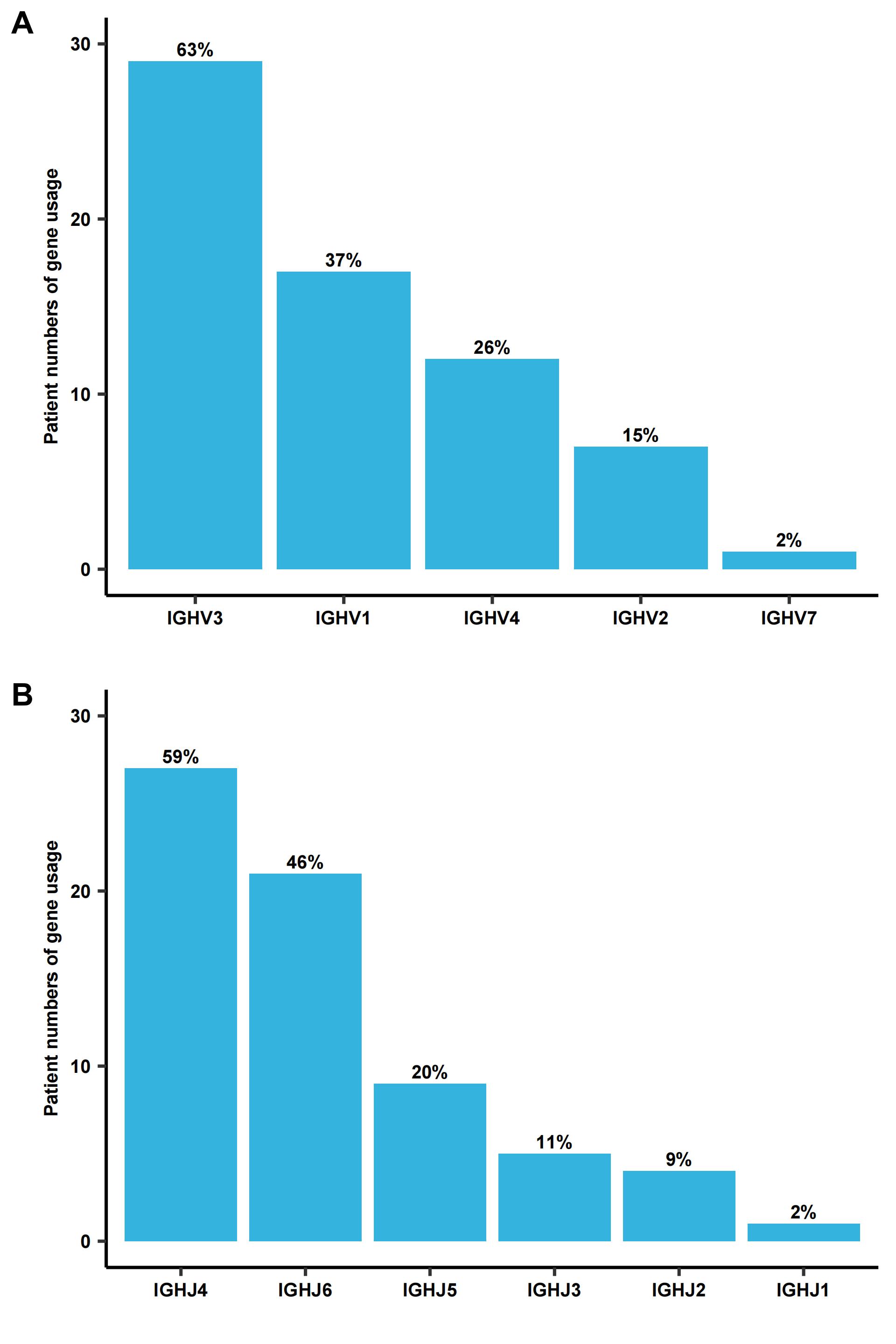


**Figure S3.** **The IGHV (A) and IGHJ (B) gene subgroup usage in the identified clonal IGH rearrangments**.


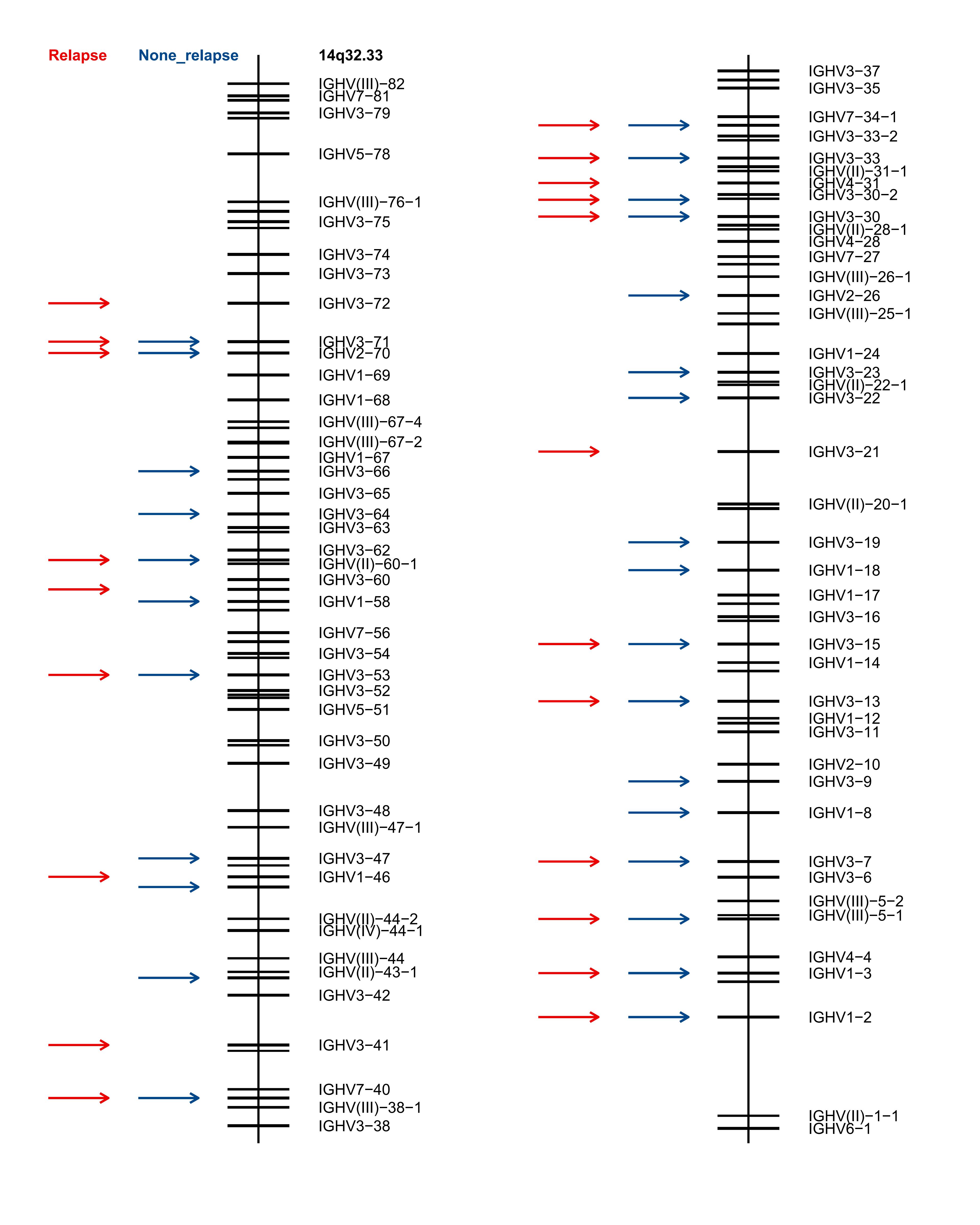


**Figure S4.** **Distribution of IGHV gene loci in relapsed versus non-relapsed patient groups.**


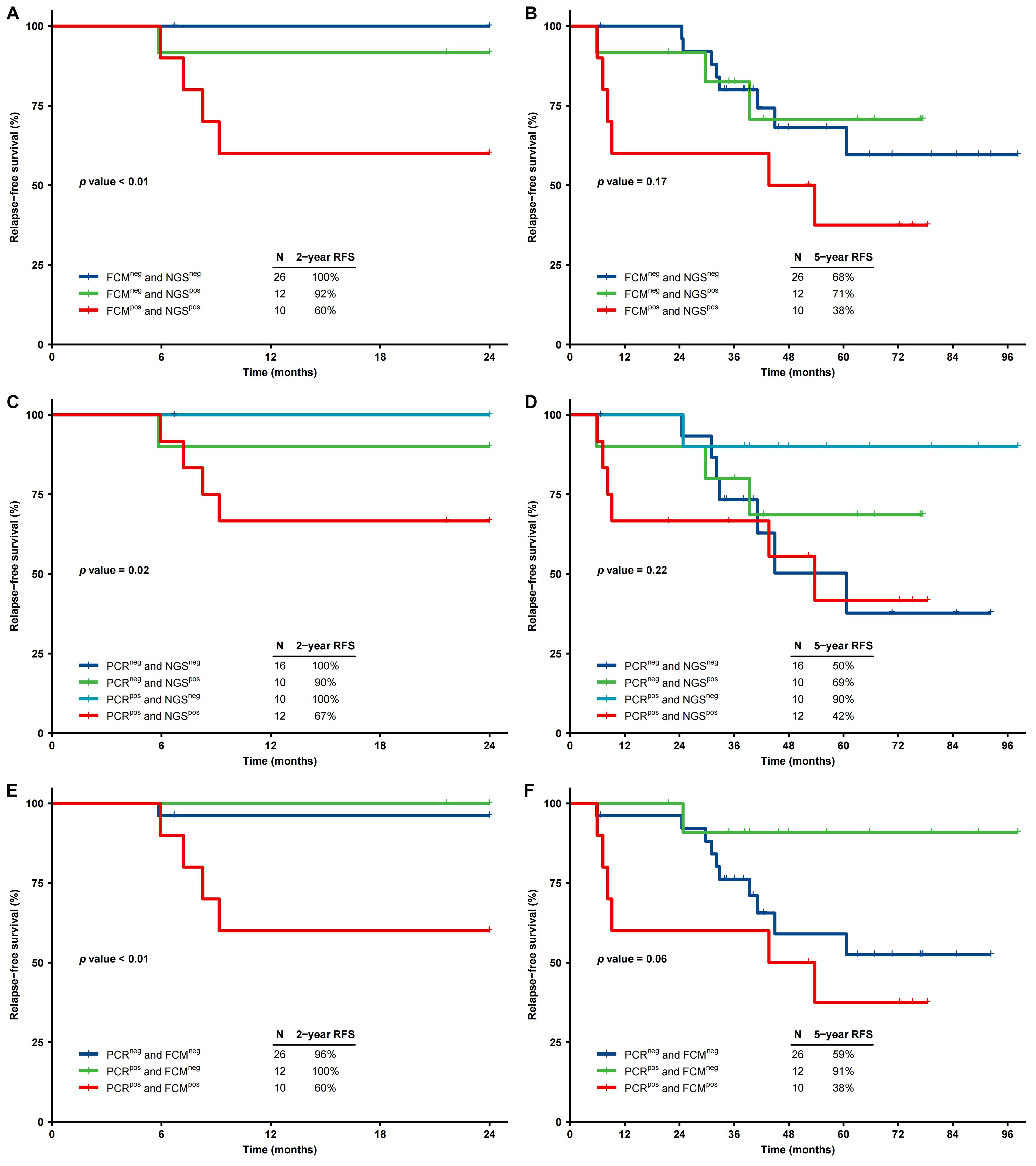


**Figure S5.** **Discondant MRD results among Ig-NGS, FCM and RT-PCR at EOI and their clinical implication**. Kaplan-Meier estimates of RFS based on MRD status by combined methods: (A) two-years RFS when combining FCM and Ig-NGS. (B) five-years RFS when combining FCM and Ig-NGS MRD. (C) two-years RFS when combining RT-PCR and Ig-NGS. (D) five-years RFS when combining RT-PCR and Ig-NGS. (E) two-years RFS when combining RT-PCR and FCM. (F). five-years RFS when combining RT-PCR and FCM.


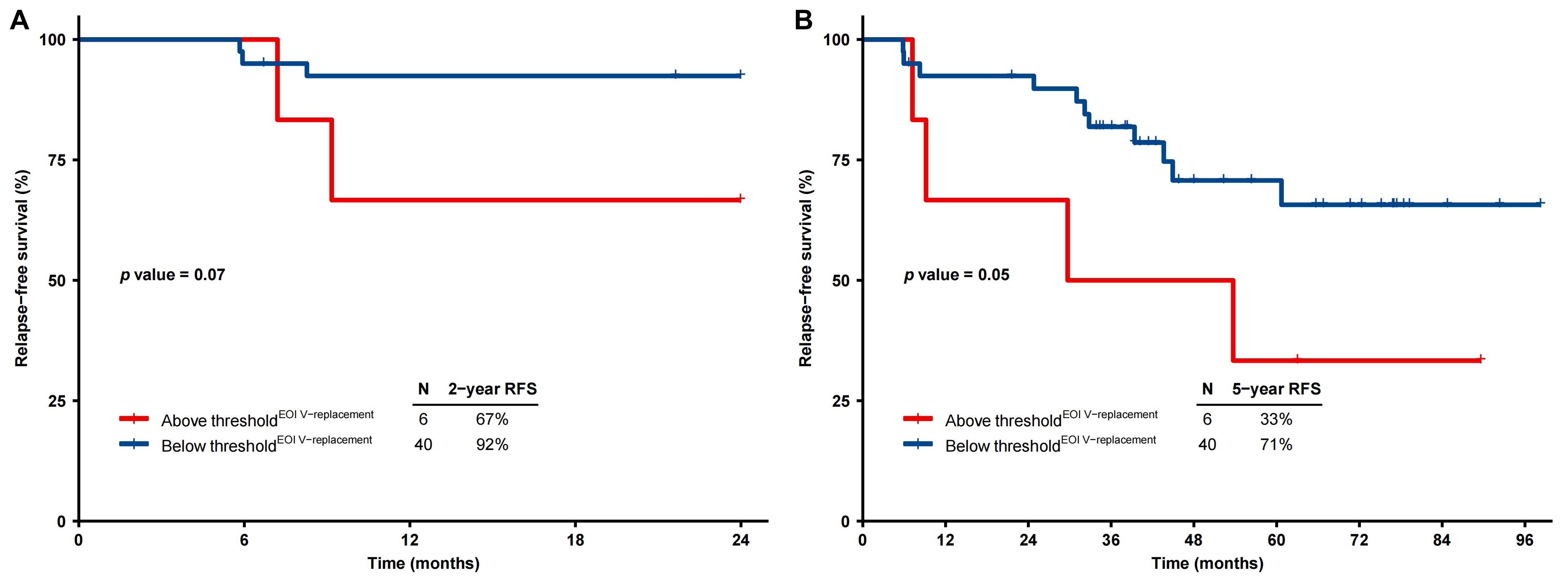


**Figure S6.** **Prognostic value of clonal evolution assessed by Ig-NGS at EOI**. Kaplan-Meier estimates of two-years (A) and five-years (B) RFS based on the number of evolved clones.
